# Laparoscopic Cholecystectomy: Some Advantages or Just an Artifice of New Technology?

**DOI:** 10.1155/1991/67569

**Published:** 1991

**Authors:** F. Penninckx, R. Aerts, R. Kerremans, P. R. Koninckx

**Affiliations:** 1 Department of Abdominal Surgery and Department of Obstetrics University Hospital Gasthuisberg Katholieke Universiteit Leuven Belgium; 2 Department of Gynaecology University Hospital Gasthuisberg Katholieke Universiteit Leuven Belgium; 3 M.D.; Department of Abdominal Surgery UZ Gasthuisberg Herestraat 49 Leuven B-3000 Belgium

## Abstract

Twelve selected patients undergoing cholecystectomy were operated in a prospective randomised study by laparoscopy (CO_2_ laser) or by classic surgery. Our preliminary results suggest that laparoscopic cholecystectomy is of clinical benefit as compared to classic cholecystectomy since it reduces the surgical trauma, limiting weight loss and shortening the hospital stay.


*HPB Surgery*, 1991, Vol. 3, pp. 291–295
Reprints available directly from the publisher
Photocopying permitted by license only

© 1991 Harwood Academic Publishers GmbH
Printed in the United Kingdom

### LAPAROSCOPIC CHOLECYSTECTOMY: SOME ADVANTAGES OR JUST AN ARTIFICE OF NEW TECHNOLOGY?

F. PENNINCKX, R. AERTS, R. KERREMANS and P.R. KONINCKX*
*Department of Abdominal Surgery and Department of Obstetrics and Gynaecology*, University Hospital Gasthuisberg, Katholieke Universiteit Leuven, Belgium*

*(Received 12 December 1989)*

Twelve selected patients undergoing cholecystectomy were operated in a prospective randomised study by laparoscopy ($CO_2$ laser) or by classic surgery. Our preliminary results suggest that laparoscopic cholecystectomy is of clinical benefit as compared to classic cholecystectomy since it reduces the surgical trauma, limiting weight loss and shortening the hospital stay.

KEY WORDS: Gall bladder, cholecystolithiasis, cholecystectomy, laparoscopy

### INTRODUCTION

Surgical procedures should be effective and safe. Moreover, total cost should be kept as low as possible both for the community and for the patient. Although mortality and morbidity rates of elective cholecystectomy (CCE) are remarkably low, there has been a proposal to reduce surgical stress by mini-trauma CCE[1]. This approach, however, is still not widely used. In 1987 laser CCE was performed through a subcostal incision. The authors claim obvious benefits although a prospective clinical trial was not performed [2]. We were impressed by the smooth convalescence in gynaecological patients after laser *laparoscopic* treatment e.g. for endometriosis, as compared to laparotomy procedures for the same pathology [3]. Therefore, we performed *laparoscopic* CCE, unaware of analogous efforts being made in other centers. The preliminary results of an on-going prospective randomized trial, evaluating the feasibility and the eventual advantages of laparoscopic CCE, are reported.

### MATERIAL AND METHODS

During May 1989, 12 non-obese women, 30-64 years old, had to undergo an elective CCE for lithiasis. Preoperative iv cholangiography, liver tests and amylasemia as well as the routine evaluation tests were normal. They were randomly

Correspondence to F. Penninckx, M.D.; Department of Abdominal Surgery, UZ Gasthuisberg, Herestraat 49, B-3000 Leuven, Belgium.

offered the possibility of laparoscopic CCE and 5 gave their informed consent. In the 7 other patients a CCE with preoperative cholangiography and manometry was performed through a limited midline incision in the upper abdomen.

Laparoscopic cholecystectomy was performed as follows. After intubation and general anaesthesia pneumoperitoneum was initiated and maintained at a pressure of 15 $cmH_20$ by the laparoflator permitting $CO_2$ lasering without visible smoke[4]. A four or five puncture technique was used: the operative laparascope with the $CO_2$ laser was introduced through the umbilicus, while three or four 5 mm trocars were introduced subcostally in the right hypochondrium. The patient was placed in the Fowler position on an antislip mattress. The gall bladder was grasped at its neck and the peritoneum at the cystic and choledochal ducts was dissected. In 3 cases the common bile duct was visualized radiologically: once by peroperative endoscopic retrogade cholangiography, once by peroperative iv cholangiography and once via a preoperatively placed nasobiliary drain. The distal end of the cystic duct was carefully identified, transsected between 2 clamps and the cystic stump was ligated twice with catgut 0 loops (Ethibinder$^R$, Ethicon). The cystic artery was coagulated by endothermia and a retrograde subserosal CCE performed with the $CO_2$ laser. At the end of the procedure any remaining fluid was removed from the subhepatic region and the pouch of Douglas. A Redon drain was left in the gall bladder bed through one of the work channels. All gall bladders could be removed intact through the umbilical incision after removal of the trocar.

Patients received piritramide (15 mg im. per injection) during the first 48 hours on request and buprenorphine (0.2 mg sublingual doses) later on. Fluid and electrolyte therapy consisted of 3 L dextrose 5% in 0.9% saline solution per day. In the absence of nausea, oral intake was started when the ileus recovered. Usually, patients are discharged on the 9th day acter CCE. However, all were informed they could leave the hospital as soon as they felt able to, provided they were in good medical condition.

### RESULTS

### *Feasibility of Laparoscopic Cholecystectomy*

CCE could be performed by laparoscopy in all 5 patients. The duration of anaesthesia was significantly longer than in the control group – 204 versus 90 minutes – but became shorter with increasing expertise. The first case took 280 minutes in contrast with 105 minutes for the 5th patient. The main problem encountered during laparoscopic CCE was related to identification and ligation of the cystic duct. In the first case a hooked absorbable clip was placed at the distal end of the cystic duct; laceration was suspected and confirmed on peroperative endoscopic cholangiography. After laparoscopic CCE a limited laparotomy was performed to ligate the duct. There were no postoperative complications. In all other patients the cystic duct was ligated twice with catgut 0 loops. In our second and third case iv. cholangiography was performed but the radiological images were not ideal. Therefore, a nasobiliary drain was placed endoscopically in our fourth case. Unfortunately this patient developed pancreatitis though of very limited clinical impact. In the fifth patient the extrahepatic duct was not visualized radiologically.

*Comparison between Laparoscopic and Surgical Cholecystectomy*

The age and weight of the patients were comparable in both groups. The only postoperative complication was a biochemical pancreatitis in 1 case after laparoscopic CCE. Some aspects of the clinical and biochemical evolution are illustrated in Table 1. In order to assess the eventual benefit of laparoscopic CCE patients 2, 3 and 5 are compared with those of the surgical CCE group. The peak value of postoperative fever was similar in both groups but the duration of postoperative fever was slightly shorter in the laparoscopic group. Patients in this group also requested somewhat less analgesics and their ileus recovered somewhat earlier than in the surgical group. The duration of hospital stay and the weight loss however were significantly reduced after laparoscopic CCE ($p<0.05$). The convalescence period was only slightly shorter after pure laparoscopy than after surgery: 29 versus 39 d. This might, however, be influenced by the social insurance system which provides and compensates for about 6 weeks off work after CCE. Changes in haematocrit, white blood cell count and serum transaminase, alkaline phosphatase and gamma glutamyl transpeptidase levels were similar in both groups. On the second day after laparoscopy serum amylase levels rose in all patients while this was observed in only 1 case after laparotomy ($p<0.001$).

### DISCUSSION

Laparoscopic cholecystectomy is feasible. Although performed in a selected group of non-obese patients without previous operation, we think that the indications for laparoscopic CCE could be extended. The duration of anaesthesia was longer for laparoscopic CCE but the surgeon had a limited experience with endoscopic surgery and the gynaecologist with CCE. As shown, the anaesthesia time significantly reduced as expertise was built up. Ligation of the cystic duct with catgut loops is rather cumbersome and the procedure will be much easier and faster if appropriate laparoscopic clips were available.

**Table 1** Postoperative evolution in women undergoing classic surgical and laparoscopic cholecystectomy

| Group | Postop. Peak (°C) | fever Duration (d) | Hosp. stay (d) | Weight change at discharge (%) | Amylase (post-vs preop) |
|---|---|---|---|---|---|
| SURGICAL (n=7) | 37.6±0.4 | 2±1.2 | 8.3±1.6 | −5.1±1.9 | ×1.1±0.5 |
| LAPAROSCOPIC | | | | | |
| Pt 1 | 38.6 | 3 | 9 | −0 | ×1.4 |
| 2 | 37.7 | 1.5 | 6 | −0 | ×3.7 |
| 3 | 37.7 | 2 | 4 | −0 | ×3.7 |
| 4 | 38.6 | 3.5 | 7 | −0.7 | ×12.8 |
| 5 | 37.4 | 1 | 4 | −5 | ×2.5 |
| Subtot. 2+3+5 | 37.6±0.2 | 1.5±0.5 | 4.7±1.2* | −1.7±2.9* | ×3.3±0.7* |

* $p<0.05$ from surgical group (Mann-Whitney test)

The clinical results indicate that surgical stress is significantly smaller after laparoscopic CCE. The shorter hospital stay and earlier recovery of the patient's general condition have important socio-economic advantages. The rise of serum amylase level after laparoscopic CCE came as a surprise. It might be related to the increased length of anaesthesia and/or the cooling of the abdominal content by $CO_2$. It might also be related to the continuously increased intra-abdominal pressure – about 15-20 cm $H_20$ – during laparoscopy. The esthetic result after laparoscopic CCE was evidently superior to that of surgery and was greatly appreciated by all patients.

In conclusion, laparoscopic CCE can be done and, with experience, will not take longer than a classic surgical CCE. The socio-economic and esthetic advantages suggest that laparoscopic CCE will become the method of choice in a number of selected patients.

### *References*

1. Moss, G. (1983) "Mini-trauma" cholecystectomy. *Journal of Abdominal Surgery*, **25**, 66–74
2. Daly, C.J. (1989) Laser cholecystectomy. In *Lasers in general surgery*, edited by S.N. Joffe, pp. 40–46. Baltimore: Williams & Wilkins
3. Gomel V. (1989) Operative laparoscopy : time for acceptance. *Fertility and Sterility*, **52**, 1–11
4. Koninckx, P.R. Demeyere, S. and Vandermeersch, E. (1988) $CO_2$-videolaser endoscopy using a new and safe insufflation apparatus which evacuates smoke continuously. *Human Reproduction*, **3 suppl. 1** 119 (abstr.)

*(Accepted by S Bengmark 18 September 1990)*

### INVITED COMMENTARY

The authors report on their early experience with laparoscopic cholecystectomy and a comparison between 5 laparoscopic and 7 conventional cholecystectomies.

In the laparoscopic group they changed to laparotomy in one case because of a possible laceration of extrahepatic bile duct system. Furthermore they saw postoperatively more mild pancreatis in the laparoscopy group.

For the comparison between laparoscopic and conventional cholecystectomy they chose the endpoints fever, hospital stay, weight change, time of operation, body image and laboratory data. It was stated that the laparoscopy cholecystectomy is beneficial in respect to the duration of hospital stay, the weight change and the esthetic results.

Conventional cholecystectomy seems to be beneficial in serum amylase and operation time. No difference occurs in the other laboratory data and postoperative fever.

Cholelithiasis is a very common disease. This is expressed in other publications like Trede[1], who reports on the results of more than 6000 conventional cholecystectomies. So the recruitment of patients is not a problem and for this the number of cases (5) is too small. Furthermore Reddick[2] (1989) reports on 25 laparoscopic versus 25 conventional cholecystectomies. Dubois (1989)[3] published over 300 cases and Perissat[4] 170 cases of laparoscopic cholecystectomy.

Between October 1989 and August 1990 we performed 250 laparoscopic cholecystectomies in our clinic and we published the results and first experience of our first 100 cases in a pilot study[5].

In this field our publication is not the first on this new technology.

We think that publishing a comparison between 5 vs 7 cases should only be allowed in special situations. For example if the disease is very rare or if it is one of the first descriptions of a new technology or if the results can change a lot.

Under such conditions the results in comparing 5 vs 7 cases of cholelithiasis does not have any relevance.

This publication presents a randomized trial, but there is no description of the clinical features of the patients. Therefore it is impossible for the reader to relate the results to his own patients.

The method of randomization is not given and there are no criteria for including or excluding a patient in or from this study.

Both operation techniques must be standardized.

For a fair comparison the operating team must be trained and experienced in both techniques. We believe that this work is not a true randomized trial.

Laparoscopic cholecystectomy is intended to be a minimally invasive or maximally atraumatic procedure. This means more comfort and less trauma for the patient. In laparoscopic cholecystectomy more comfort means:

—a fast and effective therapy
—less pain
—less fatigue
—a fast convalescence
—a better cosmetic result
—no diet

Every operation is a necessary trauma for the patient. For every trauma the body reacts on different levels and this can be summarized as "stress response". Such a stress response is transmitted by different mediators of, the endocrine system (ACTH, growth hormone, prolactin, histamine), the immune system (granulocytes) etc. and the metabolic response (glucose, lactate, albumin, acute phase protein). The response of these mediators to operation is only of relevance for the clinicians, if this response influences the outcome of the patients in terms of morbidity, complication rate or mortality.

We therefore we think, that it is very important to choose the relevant endpoints for a comparison between laparoscopic and conventional cholecystectomy.

In summary this publication is a case report of laparoscopic cholecystectomy, done by a prospective documentation of consecutive patients. It shows the feasibility of laparoscopic cholecystectomy in 5 cases with safety.

### *References*

1. Trede, M. and Schaub, W. (1990) Ein Plädoyer für Cholecystektomie. *Chirurg.* **61**, 365–369
2. Reddick, E. and Olsen, D.O. (1989) Laparoscopic laser cholecystectomy. A comparison with minilap cholecystectomy. *Surg.Endosc.* **3**, 131
3. Dubois, F., Kard, F., Berthelot, G. and Levard, H. (1990) Coelioscopic cholecystectomy. Preliminary Report of 36 cases. *Ann.Surg.* **212**, 60
4. Perissat, J., Collet, D. and Belliard, R. (1990) Gallstones: Laparoscopic treatment — cholecystectomy, cholecystostomy and lithotripsy. *Surg.Endosc.* **4**, 1–5
5. Troidl, H., Spangenberger, W., Klein, J. and Paul, A. (1990) Laparoskopische Cholezystektomie - erste Erfahrungen und Ergebnisse. *Langenbecks.Arch.* (in press)

W. Spangenberg and H. Troidl
University of Cologne
Germany

